# Few Years’ Obstetrical Practice in the Department of Oreuse

**Published:** 1853-05

**Authors:** M. Maslieurat-Lagemard


Translated for this Journal, from the Gazette des Hopitaux.

ART. IV—*Transactions of the Imperial Academy of Medicine, held at Paris, March, 22d,* 1853.

*Ten Years' Obstetrical Practice in the Department of Creuse.*—BY M. MASLIEURAT-LAGEMARD.

THE order of the day was the discussion upon the report that M. Depaul had read at a former session upon this subject.

We give extracts from this report. The paper of M Maslieurat is composed of a series of essays, which treat upon the most important questions in obstetrical practice.

The Author, classifying cases of a similar character, has divided them into nine distinct series. The first comprise six cases which were reported in a concise and clear manner. They comprise cases in which his assistance had been asked on account of the unusual length of labor. After becoming assured that it would not compromise the health of either mother or infant, he decided not to interfere with the natural powers, and the deliveries were favorable.

The cases in the second series were five—They relate to accouchments which terminated spontaneously and favorable, after having required the artificial rupture of the membranes.—Four of these were primiparæ. The fifth multiparæ. In all the dilitation was complete and the uterine contractions very feeble. The labor in the first case continued thirty hours, thirty-six in the second, and forty-eight hours in each of the other three. In all the cases the vertex presented This brief resume of the principal points which characterize his observations will suffice fully to justify his conduct, and to show that in these different circumstances he has evidently acted in conformity to sound doctrines.

The third series comprises those in which the author has had recourse to the use of ergot. The first six were primiparæ. The membranes had been ruptured for some time and the labor had already continued thirty-six or forty-eight hours. In all the uterine contractions had sensibly diminished or indeed almost entirely ceased. M. Maslieurat administered one gramme of the ergot, divided into two doses, to each of the patients. The result was most happy. In every instance the contractility of the uterus was restored, and in a few hours, without interference, all were delivered of living children. Such a result would appear to justify the conduct of the author. Nevertheless, says M. Depaul, such is not my conclusion, and in this I accord with the author himself, who, after an unfortunate experience, found his first impressions modified.

We have here a summary of the facts in the seventh case, which terminates the third series. A woman aged thirty years, well formed but of a delicate constitution, had a previous pregnancy, which presented nothing remarkable either in its progress or in its termination. She was at the full term of a second pregnancy, which had also been as natural as the first. When our author was called to her, she had been in labor forty-eight hours, and the membranes had been a long time ruptured. The pains, which had been at long intervals, and feeble from the commencment, had entirely ceased ten or twelve hours previously; the dilitation was complete, the head presented, and was fully engaged in the superior strait. The conditions appeared favorable, and he administered two doses of the ergot, of 50 centigrammes each. It was then 11 o'clock A. M.; the contractions were soon restored, and M. M. being obliged to leave, directed that they should send for him in two or three hours, if the labor was not terminated in that time.

He was sent for at 6 o'clock P. M., and as he lived at a distance of two leagues from the patient, it was seven o'clock when he arrived. The condition of the patient had very much changed during his absence; he found the countenance very much altered in appearance, the nose and lips blue, and the features horribly contracted, respiration extremely difficult, short and quick; pulse thread-like and intermittent, and so frequent that it was almost impossible to count it ; no part of the fœtus could be reached by a vaginal examination, the fingers only came in contact with a few soft, fungous masses, the nature of which could not be determined. On examination of the abdomen, the uterus was found to be contracted upon itself, and extending from the umbilicus to the pelvic excavation. In the right iliac region, a second irregular mass was discovered, nodulated, easily displaced, and which was evidently the body of the fœtus, of which the head and extremities were easily recognized.

Notwithstanding the alarming condition of the patient, the contractions were not very energetic ; and it was only three or four hours since the abdomen became painful, and the general symptoms above alluded to were first manifested. Gastrotomy, was proposed as a last resort, but the patient and her friends would not submit to the operation. The patient died in about two hours.

In the midst of the sad reflections to which this unfortunate case gave rise, and after reviewing all the favorable conditions for the use of the ergot, M. Maslieurat declared that it was impossible not to attribute to the energetic contractions produced by this drug, the rupture of the uterus ; he regretted having been obliged to leave his patient, and promised for the future to be more careful in the employment of this medicine. He alluded in closing, to the wise counsels given by our Colleague, M. Danyau, in a remarkable report read to this body.

The reporter, M. Depaul, admitting that the course pursued by M. Maslieurat was perfectly excusable, since he acted in accordance with principles generally received as correct, declares that he would condemn the ergot in those cases in which the result seemed to justify its use as well as in that which terminated so unfortunately. I have for a long time, said M. Depaul, observed the evils which occasionally result both to mother and child from the administration of this substance ; and apart from its indications in certain hæmorrhages, or in some cases of miscariage, I believe the interests of the mother, would not very much suffer by its entire suppresion.

When the labor has been prolonged to its ordinary limits, and there seem to be indications for hastening delivery, it is necessary, in choosing between augmenting the uterine contractions by the use of ergot, or when evidently insufficient for the expulsion of the fœtus, replacing them by mechanical extraction, to decide whether the inefficiency is dependent on the diminished power of the contractions or on the increased resistance. Although it is generally admitted at the present day that this medicine is indicated only in cases where the obstacles to delivery are inconsiderable, or when the contractions which may result from it will require to be continued only a short time, yet I think that all the indications which should forbid its use have not been sufficiently appreciated. One is ordinarily called to make a decision at a time when, in consequence of prolonged labor, it is possible that an injury has already resulted to the child; to interpose then an agent which increases the uterine contraction, and of which the special action is to render it permanent with exacerbations almost tetanic in their character, will be to increase very much the danger by producing a disturbance in the uterine circulation still more serious, and which may terminate in that sad mechanical injury of which I have already had occasion to speak. The evident conclusion is, that in those cases which seem best adapted to the use of the ergot, it is prudent to resort to it only after being well assured of the state of the fœtal circulation; if upon examination this is found to have already been seriously interfered with, the use of the drug will be formally contra-indicated. The forceps, which in the hands of those accustomed to their use, are ordinarily safe, ought always to have the preference.

The fourth series comprises a number, of premature labors occurring in the same woman, and which appeared to be provoked by an abnormal irritability of the skin.

Another series includes cases which required turning. In two of the cases the shoulder presented. The child was lost in both instances.

A case of symphyseotomy was the subject of the eighth series.—A woman at the end of her third pregnancy had been in labor two days when the services of M. Maslieurat were requested. Although the contractions had ceased for several hours, the patient was very much fatigued by the prolonged labor. As the membranes had been ruptured for some, time and the meconium was passing, he thought it necessary to have recourse to the forceps. The introduction of the blades was accompanied with no difficulty, but scarcely had he began to make traction than the instrument broke and he was obliged to desist. He immediately sent for one of his Colleageus, ans, who, living at some distance, arrived only after several hours The operation with the forceps was recommenced with a new instrument, but the head was found so moveable above the superior strait that after several efforts they decided to turn the child. The woman had not felt the motions of the child for a long time, and upon auscultation they were not able to hear the beatings of the fœtal heart. It was easy to introduce the hand, bring down the feet and extract it as far as the head, but this was arrested at the upper strait, and all the efforts which could be made with the hands or with the forceps were futile.

It was then determined to have recourse to symphyseotomy.—An incision was made along the median line of the sympysis, sufficiently extended to enable them to cut the cartilage, care being taken to avoid the bladder and urethra. This section was made with a blunt pointed bistoury. The separation of the bones alone was not sufficient, it was necessary to pull apart with each hand by taking hold of the anterior superior spinous process on each side. A slight noise was heard in the sacro-iliac articulations of each side, and suddenly the two branches of pubic bones were separated sufficiently to admit the finger between them ; at this moment a very light traction on the trunk of the child was sufficient to deliver the head.

Three days afterwards, this woman was taken with a chill and severe pain in the whole of the right inferior extremity. The next morning it was very much swollen, without change of color.

The feebleness of the patient would not justify a resort to energetic treatment. They contented themselves with emolient fomentations, &c., and after a short time bandages rolled moderately tight. Fifteen days afterwards, this woman was able to be up and attend to her usual labors. Since that time she had another pregnancy, which progressed naturally, and terminated spontaneously in the birth of a living child.

The reporter, in spite of the success of this operation, does not think that the course of M. Maslieurat should be followed in like cases. He would prefer embryotomy, the only procedure proper in this case.

M. Depaul closed his report by a relation of a Cæsarian operation, interesting, not only from the success which followed it, but also from the peculiar circumstances under which it was performed.

In December, 1846, M. Maslieurat was invited by three practitioners to see a patient in labor two days, and upon whom they had determined to perform this operation. The woman had a slight deformity, but had already had five pregnancies, all terminating spontaneously. It appeared upon examination that the pelvis was rather larger then otherwise, and the head although not yet engaged in the superior strait, did not seem to be of an unnatural size. He expressed his astonishment at their proposition and endeavored to induce them to make use of the forceps. They objected that that which he had taken for the head was an adherent osseous tumor, developed since the previous pregnancy. His efforts to dissuade them being useless, he determined to leave, not wishing to partake in the responsibility of such an unparalled operation; but being urgently solicited, and hoping, as he said, by his presence to restrain them from further rashness, he consented to remain, after explicitly stating his objections.

None of the three had ever performed the operation. It was undertaken by the family attendant, but scarcely had he divided the abdominal walls, than his courage failed and he was obliged to desist. It was the same with the other two, and M. Maslieurat, found himself compelled to the cruel necessity of taking the bistoury, and completing an operation which he would never have commenced. The internal wound was united by the quilled suture, the abdomen sustained by a bandage, and severe regimen prescribed: every thing progressed favorably, and in a very short time the woman was perfectly well. On examination of the pelvis, after the operation, the 'osseous tumor' of the three confreres, was nowhere to be found. The embarrassing position of M. Maslieurat, can better be imagined than described. Without the least hope of reward, since the child was already dead, he found himself compelled to complete an operation which a few minutes before he had formally condemned. The only alternative was to reunite the abdominal wound and endeavor to extract the child by the natural passages, but the most serious part of the operation, the wound of the peritoneum, was already performed.

The unlooked for success which followed this operation confirms the general reflections of the author of the memoire, upon the wonderful facility with which wounds in the departments heal, and especially in that in which he resides. If, in judging ofthe val e of hyst rotomy in general, adds the reporter, the statistics of large cities and especially of our large hospitals are taken, the conclusio arrived at will be that death to the mother is always or almost always the result. If, on the contrary, the statistics of the provinces and especially of the rural districts, are made the data, this will not be judged more dangerous then a large number of other surgical operations. The department of Creuse seems to be distinguished in late years, by constant success. In fact since 1843, the Cæsarean operation has been performed six times, and in every instance the life of the mother was saved. Three of these cases were communicated in May, 1849, by Dr. Guisard, of Gueret (vide mem. academiæ, t. xv). Two others occurred since in the practice of Dr Pezaud, of Ahun, and the sixth is the one detailed above.

M. Depaul closed the report by expressing his sense of the value of the memoire and proposing :

1st. The thanks of the Academy to M. Maslieurat for his interesting communication.

2d. That it be deposited in the archives.

3rd. That his name be entered anew upon the list of candidates for places of correspondence.

